# Supplementary material for: The effects of verbal and spatial working memory on short- and long-latency sensorimotor circuits in the motor cortex
Source: PLoS One. 2024 May 16;19(5):e0302989. doi: 10.1371/journal.pone.0302989 (PMC11098330; doi:10.1371/journal.pone.0302989)
Supplement: Attachment — Submitted filename: Attachment.docx [file pone.0302989.s002.docx]

The authors report short and long -latency afferent inhibition to probe the influence of verbal and spatial working memory load. They describe that specific sensorimotor circuits recruit, with different working memory load and probed short and long -latency afferent inhibition by PA and AP TMS-induced current.

However, there are some issues to be addressed,

1. It is not clear how the author considers TMS stimuli delivery period (i.e 1500, 1750, 2000, 2250 and 2500) during analysis. I see every conditioned was averaged. It is not clear What is the effect on SAI and LAI with different TMS stimuli delivery period. However, it was also not clear How MEP elicited during these different TMS stimuli delivery period.
2. It is difficult to understand Unconditioned or conditioned TMS stimuli, there is no clear statement.
3. It is not clear that why the author called verbal working memory, it is set of different character (shape).
4. It will be good if author interstimulus intervals (ISIs) in line 53.
5. Is there any study suggests that SAI appears to involve a thalamic projection to the motor cortex (line 75).
6. Give some reference to support the dissociation between perceptual load and working memory demands suggests some common elements across the different SAI sensorimotor circuits.
7. The line number 179 is not clear.
8. Does PA in verbal task reflect subtle differences in SAI and not in AP (SAI as well as LAI).
9. Authors mentioned every critical question to be asked in this article as limitation of this paper.
