# Supplementary material for: The effects of verbal and spatial working memory on short- and long-latency sensorimotor circuits in the motor cortex
Source: PLoS One. 2024 May 16;19(5):e0302989. doi: 10.1371/journal.pone.0302989 (PMC11098330; doi:10.1371/journal.pone.0302989)
Supplement: Attachment — Submitted filename: Lenizky and Meehan - Response to Reviewer Comments.docx [file pone.0302989.s004.docx]

**Response to Reviewer Comments**

We thank the reviewer for their additional comment. We have made the applicable revisions. Changes made to the manuscript text are highlighted in red. In our responses, specific line numbers reference the revised manuscript.

**Reviewer #2**

**Point 1.** Please rewrite the two sentences contained between lines 39-42. Afferent inhibition is not a stimulation technique. Afferent inhibition is the phenomena by which a sensory afferent volley inhibits the motor response in a given muscle and is typically studied by combining non-invasive electrical nerve stimulation with TMS over M1 (Claudia V. Turco, Jenin El-Sayes, Mitchell J. Savoie, Hunter J. Fassett, Mitchell B. Locke, Aimee J. Nelson, Short- and long-latency afferent inhibition; uses, mechanisms and influencing factors, Brain Stimulation,Volume 11, Issue 1, 2018,Pages 59-74)

*Response 1. We have the sentences in question (lines 39 to 43).*
